# Patterns of plastid gene evolution: identifying candidate genes for plastid-nuclear incompatibility across the Campanulaceae

**DOI:** 10.3389/fpls.2026.1833005

**Published:** 2026-06-16

**Authors:** Bryson Scoffield, Alfredo López-Caamal, Laura F. Galloway, Karen B. Barnard-Kubow

**Affiliations:** 1 Department of Biology, James Madison University, Harrisonburg, VA, United States; 2 Department of Biology, University of Virginia, Charlottesville, VA, United States

**Keywords:** *Campanula americana*, Campanulaceae, coevolution, dN/dS, plastid genome evolution, plastid translation, plastid-nuclear incompatibility, purifying selection

## Abstract

Most protein complexes within the chloroplast consist of both plastid and nuclear-encoded subunits. This integration leads to potential coevolution between the genomes, with disruption of coevolution a potential cause of plastid-nuclear incompatibility (PNI). Both plastid-nuclear coevolution and PNI have been found across many, sometimes overlapping, lineages of flowering plants. However, drawing a direct connection between these two phenomena has been difficult as the underlying genetics of PNI have been determined in only a handful of cases. The goal of this study was to identify candidate plastid genes contributing to the PNI observed in *Campanula americana* by analyzing patterns of evolution in plastid genes from the two most divergent lineages of *C. americana* as well as species across the Campanulaceae. Photosynthetic genes exhibited low rates of nucleotide substitution and dN/dS ratios indicating purifying selection, while several genes/gene families related to gene regulation and proteostasis (*rps*, *clpP1*, *ycf2*) exhibited elevated rates of nucleotide substitution and dN/dS ratios indicating relaxed or positive selection. The *rps* genes are the most likely candidates for contributing to the PNI in *C. americana* due to their high dN/dS ratios in both *C. americana* lineages and an increased rate of sequence evolution in *C. americana* and its close relatives. These results suggest that disruption of plastid translation could be a mechanism for intraspecific PNI in this species.

## Introduction

Plastids are essential for plants as they carry out a variety of metabolic processes, with photosynthesis the most well-known. Plastids originated as a cyanobacterial endosymbiont with a prokaryotic like genome but now exhibit a greatly reduced genome due to most genes having been lost or transferred to the nucleus (Sierra et al., 2023; Timmis et al., 2004; Wicke et al., 2011). Accordingly, most proteins required for plastid function are now encoded by the nuclear genome and transported back into the plastids (Gould et al., 2008; Knopp et al., 2020). These nuclear-encoded plastid-targeted proteins must closely interact with the remaining plastid-encoded proteins to carry out essential processes such as photosynthesis, gene regulation, and protein homeostasis. This close interaction is hypothesized to lead to plastid-nuclear coevolution, where mutations arising in a gene, whether plastid or nuclear, lead to selection for compensatory mutations in the interacting subunits encoded by the other genome (Sloan et al., 2018). Such signatures of plastid-nuclear coevolution have been observed across multiple species of flowering plants (Forsythe et al., 2021; Postel et al., 2022; Rockenbach et al., 2016; Sloan et al., 2014b; Weng et al., 2016; Williams et al., 2019; Zhang et al., 2015).

While plastid-nuclear coevolution ensures the maintenance of well-matched plastid and nuclear proteins within a population or genetic lineage, and thus proper functioning of the plastid, it simultaneously increases the probability of mismatched proteins in between lineage hybrids. This mismatch may lead to reduced plastid function, for example photosynthetic ability, and thus a decrease in fitness. Accordingly, the disruption of plastid-nuclear coevolution could be a major contributor of genetic incompatibility and thus reproductive isolation in flowering plants (Bogdanova, 2020; Greiner et al., 2011; Postel and Touzet, 2020).

In fact, such plastid-nuclear genetic incompatibility (PNI) has been observed across multiple families of angiosperms (e.g. Campanulaceae, Caryophyllaceae, Ericaceae, Fabaceae, Geraniaceae, Onagraceae, Passifloraceae, Solanaceae; Barnard-Kubow et al., 2016; Breman et al., 2020; Greiner et al., 2011; Mráček, 2006; Postel et al., 2022; Schmitz-Linneweber et al., 2005; Ureshino et al., 1999), where it typically manifests as chlorosis and albinism (occasionally reduced pollen fertility, Bogdanova et al., 2015). However, drawing a direct link between these examples of PNI and plastid-nuclear coevolution is difficult, as in most cases the genetics underlying PNI remain unknown. In addition, in the few cases where the genetics have been determined, the contributing genes and mechanism have differed, ranging from RNA editing (Schmitz-Linneweber et al., 2005), to fatty acid biosynthesis (Bogdanova et al., 2015), to operon regulation (Zupok et al., 2021). Thus, it remains unclear whether the generation of plastid-nuclear coevolution, its subsequent disruption, and the potential resulting PNI follow predictable paths, driven by similar genes and mechanisms across lineages.

Plastid-nuclear coevolution and subsequent potential PNI is likely to be particularly prominent in taxa with rapidly evolving plastid genomes. Most plastid genomes in angiosperms are highly conserved in sequence, gene order and content (Jansen et al., 2007; Wicke et al., 2011). However, multiple independent lineages of angiosperms demonstrate accelerated plastid genome evolution, often in a common subset of genes, including elevated rates of nucleotide substitution and dN/dS ratios (Barnard-Kubow et al., 2014; Greiner et al., 2008; Guisinger et al., 2008; Gurdon and Maliga, 2014; Jansen et al., 2007; Shrestha et al., 2019; Sloan et al., 2014a; Weng et al., 2016). These lineages also often contain species in which PNI has been documented (Barnard-Kubow et al., 2016; Breman et al., 2020; Greiner et al., 2011; Mráček, 2006; Postel et al., 2022), making them ideal model systems for investigating the mechanistic link between cytonuclear-coevolution and PNI.

*Campanula americana* is a monocarpic herb in the Campanulaceae that exhibits both accelerated plastid genome evolution and PNI (Barnard-Kubow et al., 2016; Barnard-Kubow et al., 2014). PNI has been well characterized with crossing between divergent genetic lineages leading to chlorosis and an associated reduction in survival of up to 80% (Barnard-Kubow et al., 2016). The genetics underlying PNI in *C. americana* remain unknown, but an analysis focused on a single genetic lineage found several plastid genes/gene families with increased nucleotide substitution rates and elevated dN/dS ratios, specifically *ycf1*, *ycf2*, *clpP*, and the small ribosomal proteins (*rps*) (Barnard-Kubow et al., 2014). A similar set of genes exhibited elevated sequence divergence among genetic lineages of *C. americana* including the small and large ribosomal proteins (*rps* and *rpl*) as well as *ycf1(Tic214)* and *ycf2* (López-Caamal et al., 2025). These results suggest that PNI in *C. americana* may be associated with gene translation (ribosomal proteins), protein import (*ycf1*, *ycf2*) or protein turnover (*clpP*).

In this study we further investigate the genetics underlying the plastid component of PNI in *C. americana* in two ways. First, we use plastid genomes from divergent genetic lineages of *C. americana* to investigate variation in rates of evolution and patterns of selection among plastid genes. Second, we expand this analysis to include the broader context of the Campanulaceae, a family that exhibits dynamic plastid genome evolution (Cosner et al., 2004; Haberle et al., 2008; Knox, 2014), by including species from across the family at varying phylogenetic distances from *C. americana*. We then ask: 1) Does the same set of genes exhibit signatures of accelerated evolution and differentiation in selection across multiple lineages of *C. americana*? 2) How have these genes evolved across the Campanulaceae? 3) Which plastid genes appear most relevant to PNI in *C. americana*?

## Materials and methods

Annotated plastid genomes from across the *Campanulaceae* and several outgroups were obtained from NCBI’s Genbank as well as from previous studies (López et al., 2025; **Table 1**). In addition, representative genomes from two of *C. americana*’s three lineages were used for analysis (Western, Appalachian). While all three *C. americana* lineages were initially included, the Eastern lineage was dropped due to high similarity with the Western plastid genomes. Three out-groups varying in phylogenetic distance from the Campanulaceae were also used. *Carpodetus serratus* is a member of the Rousseaceae, sister to the Campanulaceae. *Helianthus annuus* is in the order Asterales with the Campanulaceae, and *Nicotiana tabacum* in the order Solanales is the most distant.

**Table 1:** Species used in this study for comparison of dN/dS ratios, ordered by increasing phylogenetic distance from *Campanula americana*.

Species	GenBank ID:	Family
*Campanula americana Appalachian*	KJ920499.1-KJ920507.1	Campanulaceae
*Campanula americana Western*	PQ523296	Campanulaceae
*Triodanis perfoliata*	PQ526809	Campanulaceae
*Asyneuma japonicum*	OR805474	Campanulaceae
*Adenophora racemosa/pereskiifolia*	MT012303	Campanulaceae
*Adenophora divaricata*	NC_036221	Campanulaceae
*Hanabusaya asiatica*	NC_024732	Campanulaceae
*Trachelium caeruleum*	NC_010442	Campanulaceae
*Campanula takesimana*	NC_026203	Campanulaceae
*Campanula pallida*	NC_063742	Campanulaceae
*Jasione montana*	OZ060881	Campanulaceae
*Wahlenbergia marginata*	NC_063740	Campanulaceae
*Codonopsis bhutanica*	NC_063738	Campanulaceae
*Cyananthus lobatus*	NC_063739	Campanulaceae
*Platycodon grandiflorus*	NC_035624	Campanulaceae
*Carpodetus serratus*	NC_036084	Rousseaceae
*Helianthus annuus*	NC_007977	Asteraceae
*Nicotiana tabacum*	NC_001879	Solanaceae

All protein-coding plastid gene sequences were extracted and aligned using the software Geneious Prime (Geneious Prime 2025.03.24; https://www.geneious.com). The coding sequence for each gene was extracted from the annotated genome files, ensuring genes located in the inverted repeat region were only used once. Gene alignments were generated using a combination of alignment algorithms available in Geneious, including Geneious, MUSCLE, and translational. Then the alignments were manually curated to remove un-alignable regions and stop codons. Finally, gene alignments were concatenated by gene family as the number of SNPs in a single gene was frequently insufficient for analysis (**Table 2**). For *clpP1*, *Platycodon grandiflorus* was so divergent its sequence was dropped from the alignment. In addition, *ycf1* was so divergent between species that it proved impossible to align and was therefore not included in this study.

**Table 2:** Gene families for which species alignments were concatenated, their functional category, and the number of genes in each family.

Category	Gene family	#Genes	Total length (bp)
Photosynthesis	*atp*	6	4944
	*ndh*	11	10419
	*paf*	2	1080
	*pet*	6	2382
	*psa*	5	4935
	*psb*	13	6411
	*rbcL*	1	1426
Gene regulation	*rpo*	4	10530
	*rpl*	8	3069
	*rps*	12	5841
Proteostasis	*clpP1*	1	687
	*ycf2*	1	4578
Other	*ccsA*	1	954
	*cemA*	1	690
	*matK*	1	1593

Total length refers to the length of each concatenated alignment.

**Figure 1:**
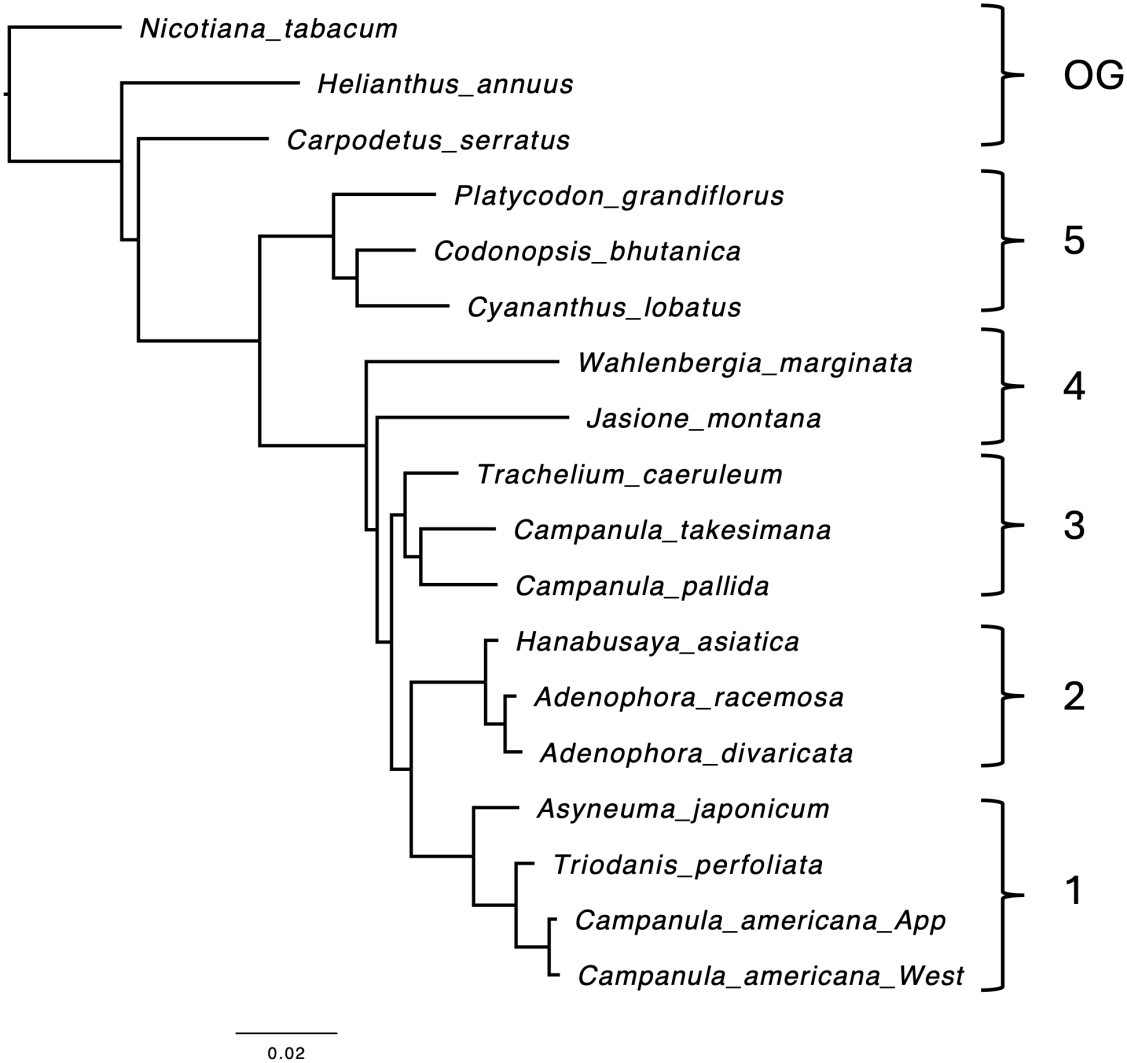
Phylogenetic tree of the taxa used for the dN/dS analyses which was constructed in RaxML with 1000 bootstraps. An unrooted version of this tree topology was used as the constraint tree for all PAML analyses. The labels refer to the clade groupings used in the PAML branch models, with 1 being the focal clade containing *C. americana*, and the numbers increasing with distance from *C. americana*. While clade 4 is not strictly a clade, we refer to it as such when describing the PAML analyses for simplicities sake. OG, outgroups. Tree was visualized in FigTree (v1.4.4). Branch support values are not shown but were all 100%.

All gene alignments, except for *clpP1*, were concatenated to form an overall alignment from which we generated a consensus phylogenetic tree to use as input for Phylogenetic Analysis by Maximum Likelihood (PAML; Yang, 2007). The consensus tree was constructed using the RAxML plugin in Geneious with the rapid bootstrapping and subsequent ML search algorithm, GTR+GAMMA nucleotide model and 1000 bootstraps. The resulting unrooted output tree was used as the input tree file for all PAML analyses. The output tree was then rooted for visualization in FigTree (v1.4.4; https://github.com/rambaut/figtree/) using *N. tabacum* as the outgroup (**Figure 1**).

PAML was used to analyze the plastid gene alignments and estimate dN, dS, and dN/dS for each branch in the tree, where dN is the nonsynonymous substitution rate and the dS is the synonymous substitution rate. Codon frequencies were determined by an F3x4 model. The parameter values for dN/dS and the transition/transversion ratio were estimated from the data with initial values of 0.4 and 2, respectively. Separate dN/dS values were estimated for each branch using model 1. In some cases, dN or dS was estimated as zero, making dN/dS not calculatable. In these cases, dN/dS was marked as NA and excluded from analysis and visualization.

To determine whether dN/dS ratios varied depending upon gene functional category and/or species, a linear model was run to determine whether dN/dS was significantly influenced by gene category (photosynthesis, gene regulation, proteostasis, other; **Table 2**) and/or species using only the dN/dS values for the tips. The model was run using the glmmTMB function (Brooks et al., 2017) in R (v. 4.4.3; R Core Team, 2025) via RStudio (v. 2024.12.1.563; Posit team, 2025) with both gene category and species as main effects and gene family treated as a random effect nested within gene category. dN/dS was log transformed for the analysis due to the data being right skewed, The conditional and marginal R^2^ values were calculated using the r2_nakagawa function from the performance package, and the intraclass correlation coefficient was calculated using the icc function (Lüdecke et al., 2021; Nakagawa et al., 2017).

For genes/gene concatenations that exhibited a dN/dS value of above 1 in at least one of the two *C. americana* lineages, branch models were carried out using PAML to investigate the phylogenetic pattern of dN/dS across the Campanulaceae. First, models were run with the same parameters as above, but using model=0, such that one dN/dS value is estimated across the entire tree. Second, models were run where a subset of tips were assigned to a target (foreground) group and compared to the remaining tips and branches. Using model=2, two separate dN/dS values were estimated, one for each group. As these models are nested within the model 0, likelihood ratio tests (LRT) were used to determine if the models with 2 dN/dS values were a significantly better fit than the model with only one dN/dS value. If more than one model with 2 dN/dS values exhibited significantly better fits than model 0, the model with the best log likelihood value was determined to be the best model. This best model was then compared to models with 3 groups and 3 dN/dS values to determine if these models were a significantly better fit. In each analysis, taxa were assigned to the target group at the clade level, where all taxa within a clade were either included or excluded from the target group(s).

Elevated dN/dS ratios, particularly those above one, are often interpreted as evidence for positive selection, but can be caused by other factors included relaxed purifying selection, and genetic drift (Wang et al., 2024). To address this issue, further investigation of positive selection acting on some plastid genes was carried out using the HyPhy methods BUSTED (Branch-Site Unrestricted Statistical Test for Episodic Diversification (Murrell et al., 2015)) and aBSREL (adaptive Branch-Site Random Effects Likelihood (Smith et al., 2015)). First, all genes from the photosynthesis, gene regulation, and the other functional categories (**Table 2**) were run through BUSTED testing for signatures of episodic diversifying selection across the entire phylogeny, excluding outgroups. If there was evidence for episodic diversifying selection, BUSTED was then run on each clade individual (1-5). Finally, if BUSTED then identified evidence of episodic diversifying selection in clade 1, aBSREL was used to test for positive selection on each branch within Clade 1.

Rates of plastid gene evolution across time were examined by optimizing branch lengths on the constrained rooted tree generated from the concatenated alignments. Each gene/gene family alignment was used to optimize branch length on the constrained input tree using the “evaluate” command in RAxML-NG (v1.2.2; (Kozlov et al., 2019) with a GTR+G model of evolution and the setting the brlen command to scaled. Due to the high similarity among the photosynthetic genes/gene families these were further concatenated and run as one alignment/tree. Trees were visualized and standardized by scale using FigTree (v1.4.4).

## Results

**Figure 2:**
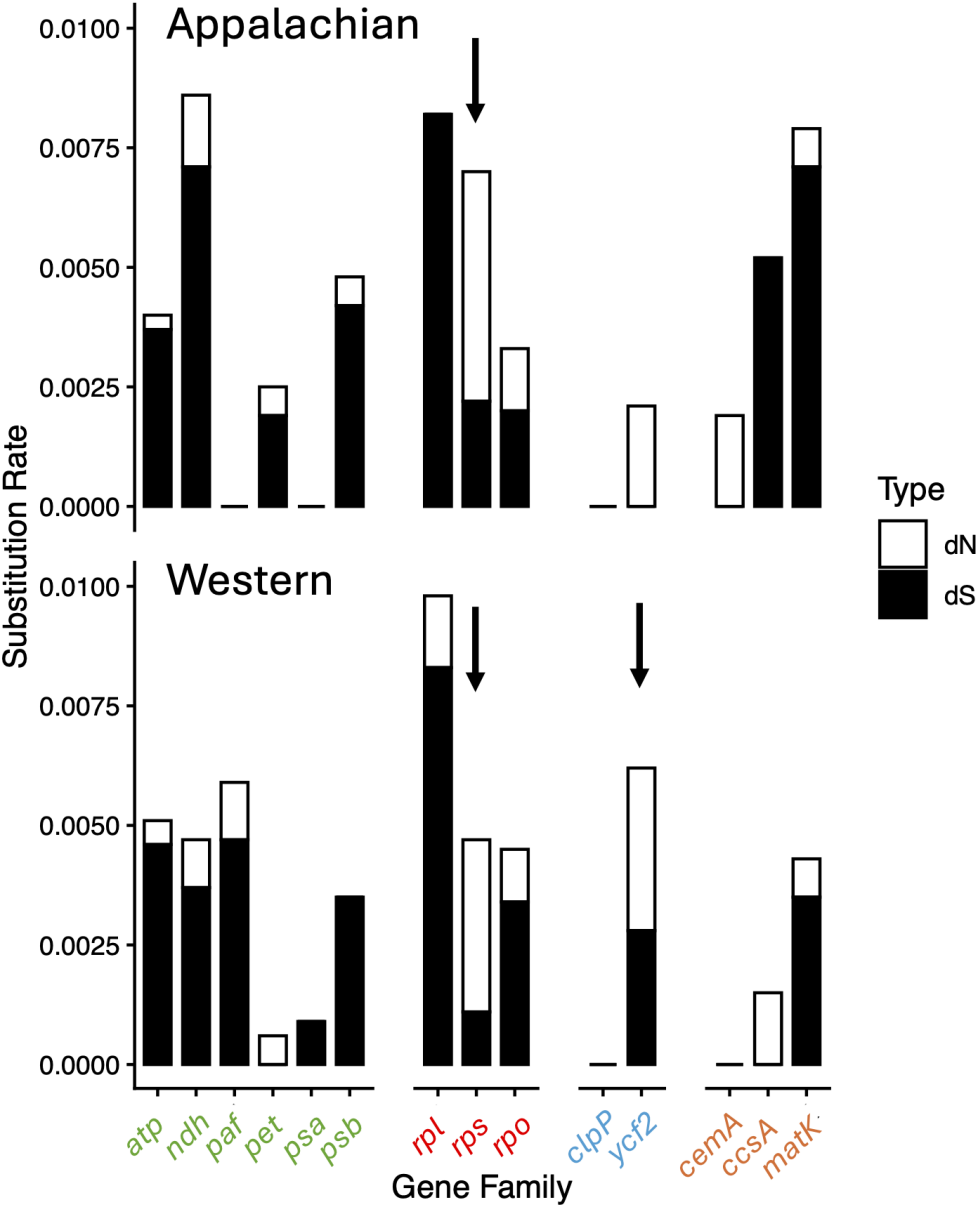
Rates of non-synonymous (dN) and synonymous (dS) substitutions on the branches leading to the Appalachian and Western lineages of *Campanula americana*. Genes are grouped according to functional category: photosynthesis, gene regulation, proteostasis, and other from left to right. Arrows indicate genes/gene families where the dN/dS ratio was above one. For genes/gene families where either dN or dS was zero, dN/dS was uncalculatable.

Within *C. americana*, most of the genes/gene families exhibited much lower rates of nonsynonymous substitutions (dN) than synonymous (dS) on the branches leading to the Appalachian and Western lineages (**Figure 2**), leading to low dN/dS ratios. This pattern was particularly pronounced for photosynthesis-related genes. One exception were the *rps* genes, which exhibited a greater value of dN in both lineages, leading to a dN/dS ratio of above one. Elevated dN was also observed for *ycf2* in both lineages, though a dN/dS above one was only observed in the Western lineage, as dS was zero in the Appalachian lineage making the ratio incalculable.

For both the *rps* genes and *ycf2*, the branch leading to the *C. americana* lineages did not exhibit dN/dS ratios above one, though for the *rps* genes the ratio was elevated (0.82). Two other genes previously associated with elevated dN/dS in *C. americana*, *clpP1* and *ycf1*, exhibited dN and dS values of zero (*clpP1*) or were not included in the analysis due to high levels of divergence leading to un-alignable sequences (*ycf1*).

**Figure 3:**
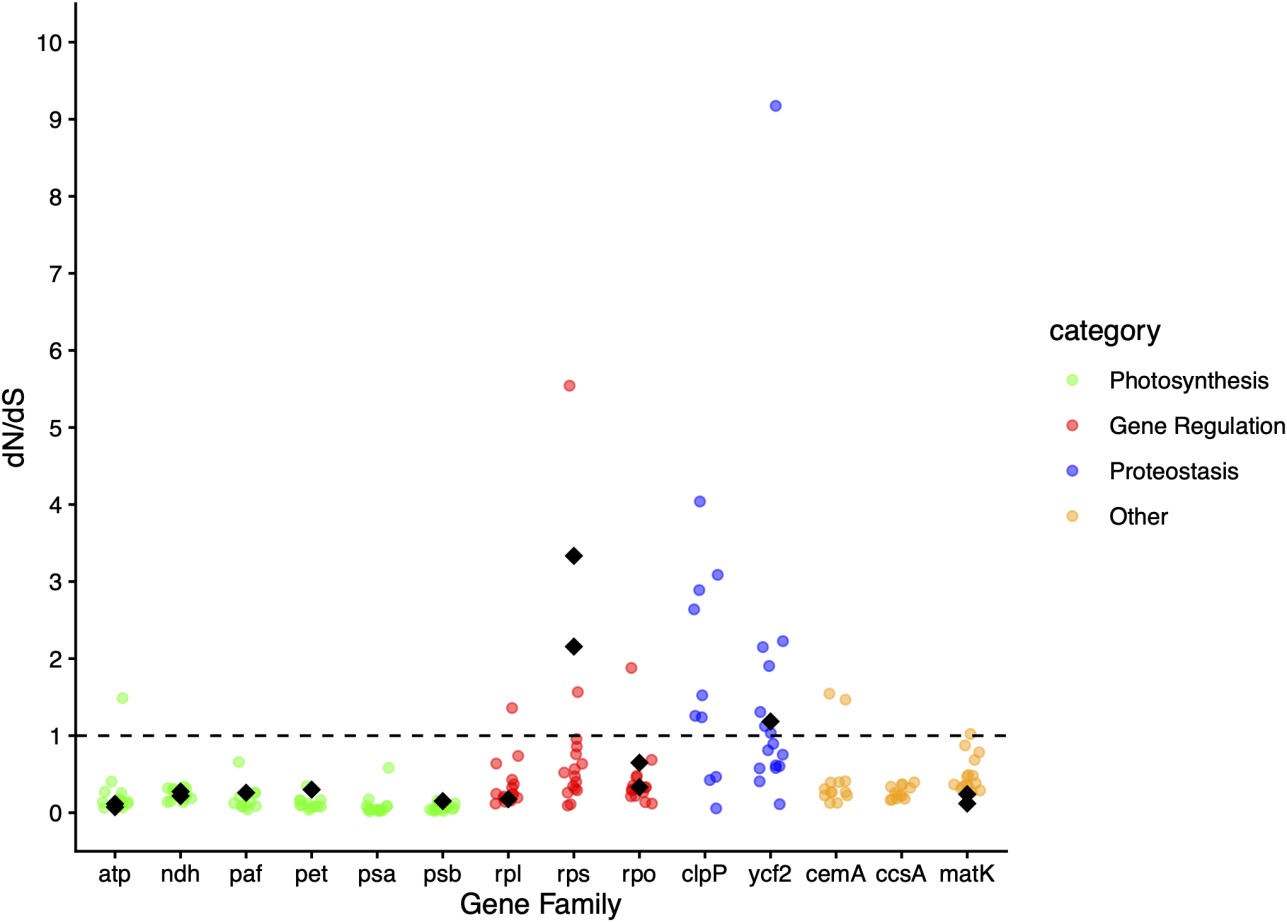
dN/dS ratios for species in the family Campanulaceae as well as the three outgroups separated by gene family. Points are colored according to function. Black diamonds are the dN/dS ratios for two *C. americana* lineages (Appalachian, Western). The dashed line indicates a dN/dS ratio of 1.

Across all species, dN/dS ratios varied among gene families with a significant effect of functional category (df=3, F = 44.05, p<0.001; **Figure 3**). Genes related to gene regulation (z=3.95, p<0.001), proteostasis (z=6.31, p<0.001), and other (z=2.81, p=0.005) all exhibited higher dN/dS ratios than photosynthetic genes. The model explained a substantial amount of variation in dN/dS with a conditional and marginal R^2^ of 0.688 and 0.593 respectively. The proportion of variance explained by the random effect of gene/gene family explained a smaller amount of variation with an unadjusted and adjusted intraclass correlation coefficient (ICC) of 0.095 and 0.233 respectively. The photosynthetic genes almost all had dN/dS values well under one, while proteostasis, gene regulation and other genes exhibited elevated dN/dS values, with some at or above one (**Figure 3**). The genes/gene families most consistently exhibiting elevated dN/dS ratios were *ycf2*, *clpP1*, and *rps*. The dN/dS ratios for *ycf2* are likely an underestimate, as large sections of un-alignable sequence had to be removed.

**Figure 4:**
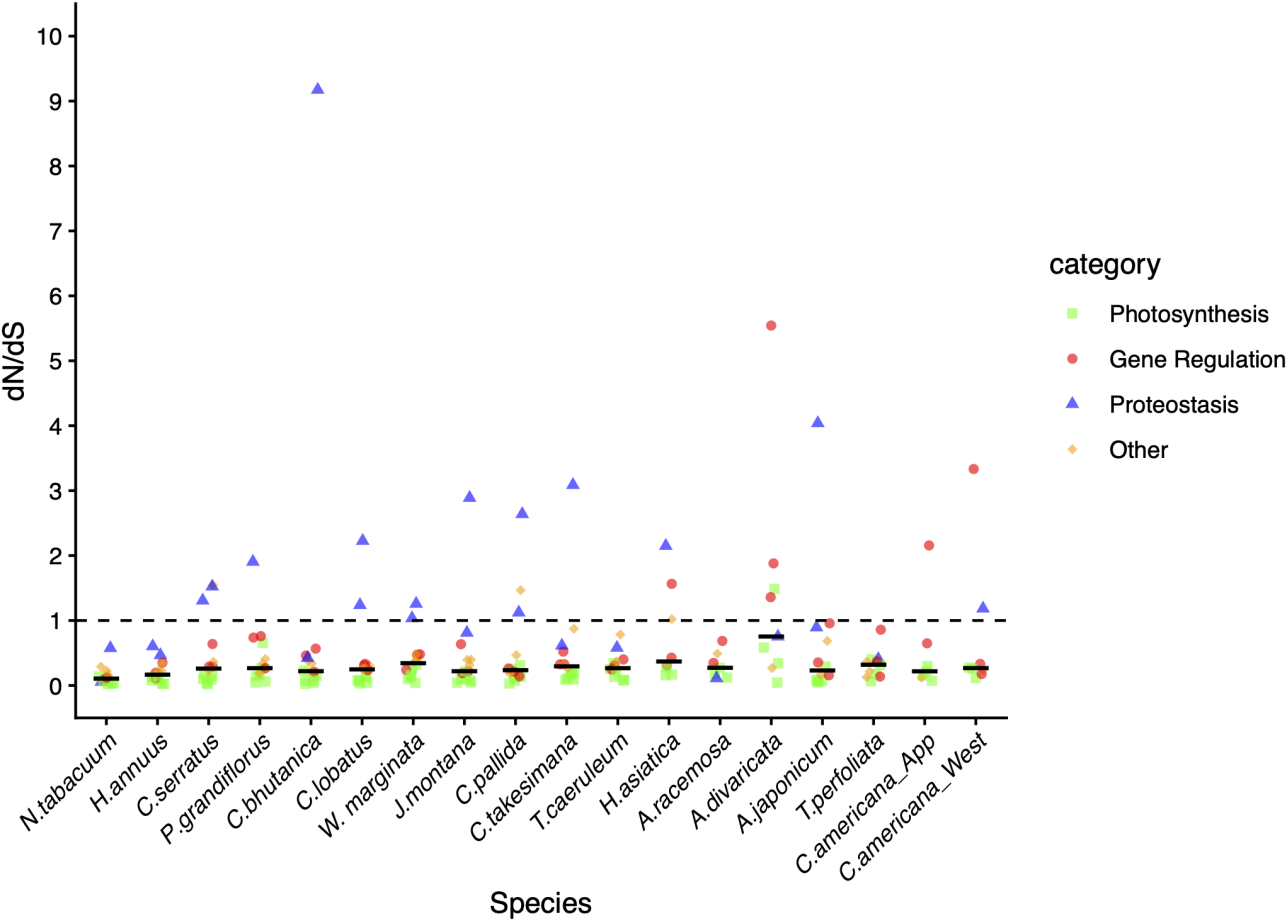
dN/dS ratios of gene families separated by species. Points are colored according to function. Black bars indicate the median dN/dS. The dashed line indicates a dN/dS ratio of 1. Species are ordered by increasing phylogenetic distance from *C. americana*.

Ratios of dN/dS also varied significantly between species (df=17, F = 83.45, p<0.001; **Figure 4**). Compared to *Nicotiana tabacum*, all the Campanulaceae species exhibited elevated dN/dS ratios, even after correcting for multiple tests (corrected p-value: 0.05/17 = 0.003). *Adenophora divaricata* had the strongest pattern with a median dN/dS of 0.754 (**Figure 4**). Even *Carpodetus serratus*, in the family sister to Campanulaceae, exhibited significantly elevated dN/dS ratios relative to *N. tabacum* (z=3.78, p<0.001).

There were distinct patterns of elevated dN/dS across the Campanulaceae depending on functional category and gene/gene family (**Figure 4**). For example, genes involved in gene regulation only exhibited dN/dS values above one in clade 1 (focal clade containing *C. americana*) and 2 (sister to the focal clade). This was driven primarily by the *rps* genes which exhibited a dN/dS ratio above one in four taxa in these clades, including both *C. americana* lineages. The *rpl* and *rpo* genes only exhibited dN/dS values above one in *A. divaricata*, the species with the highest values of dN/dS. In contrast, genes involved with proteostasis, *ycf2* and *clpP1*, exhibited dN/dS values above one across clades, yet only *ycf2* exhibited a dN/dS value above one in *C. americana*, and then only in one lineage.

Further investigation of the *rps* genes and *ycf2*, the genes/gene families with dN/dS above one in at least one *C. americana* lineage, reinforced the distinct patterns of evolution across the Campanulaceae. Branch models in PAML found that the best model for the *rps* genes was a model in which tips were assigned to two groups (**Table 3A**), one containing the focal (1) and sister clades (2) with an estimated dN/dS of 1.36, and the other containing the outgroups and remaining three clades (3-5) with an estimated dN/dS of 0.49.

**Table 3:** Using branch models to investigate patterns of evolution of the (A) *rps* and (B) *ycf2* genes across the Campanulaceae.

A. *rps*								
Model	Groups compared	NP	lnL0	Focal group dN/dS	Model compared to	df	2Δℓ	p
m0	none	35	-20392.4083	0.5058				
m2	OG vs 5,4,3,2,1	36	-20389.5940	0.5745	m0	1	5.6287	0.0177*
m2	OG, 5 vs 4,3,2,1	36	-20389.6562	0.5965	m0	1	5.5042	0.0190*
m2	OG,5,4 vs 3,2,1	36	-20390.6851	0.6320	m0	1	3.4463	0.0634
**m2**	**OG,5,4,3 vs 2,1**	**36**	**-20381.4767**	**1.3664**	**m0**	**1**	**21.8632**	<0.0001***
m2	OG,5,4,3,2 vs 1	36	-20385.1701	1.3297	m0	1	14.4763	0.0001***
m2	OG,5,4,3 vs 2 vs 1	37	-20381.4635	1.3317	m2 OG,5,4,3 vs 2,1	1	0.0263	0.8711
m1		67	-20240.4609		m2 OG,5,4,3 vs 2,1	31	282.0314	<0.0001***
B. ycf2								
m2	*C.bhutanica* vs OG,5,4,3,2,1	36	-22012.7931	1.0910				
m2	*C.bhutanica* vs OG vs 5,4,3,2,1	37	-22007.557	0.9300	*C.bhutanica* vs OG,5,4,3,2,1	1	10.4717	0.0012**
**m2**	***C.bhutanica* vs OG,5 vs 4,3,2,1**	**37**	**-22003.1912**	**0.8638**	***C.bhutanica* vs OG,5,4,3,2,1**	**1**	**19.2038**	<0.0001***
m2	*C.bhutanica* vs OG,5,4 vs 3,2,1	37	-22007.4470	0.7957	*C.bhutanica* vs OG,5,4,3,2,1	1	10.6922	0.0011**
m2	*C.bhutanica* vs OG,5,4,3 vs 2,1	37	-22011.5768	0.7800	*C.bhutanica* vs OG,5,4,3,2,1	1	2.4326	0.1188
m2	*C.bhutanica* vs OG,5,4,3,2 vs 1	37	-22012.4066	0.8828	*C.bhutanica* vs OG,5,4,3,2,1	1	0.7730	0.3793
m2	*C.bhutanica* vs OG,5 vs 4 vs 3,2,1	38	-22002.5601	0.7905	*C.bhutanica* vs OG,5 vs 4,3,2,1	1	1.2620	0.2613
m2	*C.bhutanica* vs OG,5 vs 4,3 vs 2,1	38	-22003.0265	0.7684	*C.bhutanica* vs OG,5 vs 4,3,2,1	1	0.3294	0.5660
m2	*C.bhutanica* vs OG,5 vs 4,3,2 vs 1	38	-22003.1909	0.8683	*C.bhutanica* vs OG,5 vs 4,3,2,1	1	0.0005	0.9817
m1		67	-21963.5323		*C.bhutanica* vs OG,5 vs 4,3,2,1	30	79.3177	<0.0001***

Model 0 (m0) estimates one dN/dS ratio across the entire tree. Model 2 (m2) allows different dN/dS values to be assigned to 2 groups by the user. NP= number of parameters. Focal group is the group in the comparison containing *C. americana*. The best fitting model in terms of lowest log-likelihood and with no significantly better nested model is in bold. Model 1 (m1), where dN/dS is estimated for all branches separately is included for comparison.

The *ycf2* branch models were complicated by an outlier for *Codonopsis bhutanica* (dN/dS = 9.17). Here, the model only ran if *C. bhutanica* was put it its own group (model 0, with a single dN/dS value across the tree did not run and was excluded). Thus, models were compared to one in which *C. bhutanica* was placed in its own group relative to the rest of the tree. Here, the best model had three groups (**Table 3B**): one with *C. bhuntanica* (dN/dS = 9.34), a second with the outgroups and the remaining two clade 5 taxa (dN/dS = 1.31) and a third with clades 1-4, including *C. americana* (dN/dS = 0.86).

Tests for positive selection using branch-site models in HyPhy examined all chloroplast genes. However, only the *rps* genes, *clpP*, *ycf2*, and *matK* showed evidence of episodic positive selection (**Table 4**). Among these, *matK* showed no evidence for episodic positive selection in any individual clade, while *rps*, *clpP* and *ycf2* all exhibited signatures of episodic positive selection in multiple individual clades, including Clade 1. Within Clade 1, the *rps* genes were the only genes that exhibited a signature of positive selection on the branch leading to *C. americana*, as well as to the Western, but not Appalachian, *C. americana* lineage.

**Table 4:** Using branch-site models BUSTED and aBSREL to test for signatures of positive selection in select plastid genes/gene families.

A. HyPhy BUSTED test for signatures of positive selection				
Model	rps	clpP	ycf2	matK
Full Tree	0.0258*	<0.001***	<0.001***	0.021*
Clade 5 Full	0.0215*	0.002**	<0.001***	0.500
Clade 4 Full	0.498	<0.001***	<0.001***	0.412
Clade 3 Full	0.094	<0.001***	**0.026**	0.500
Clade 2 Full	0.0327*	0.0172*	0.110	0.364
Clade 1 Full	<0.001***	0.003**	**0.037**	0.500
B. HyPhy aBSREL test for signatures of positive selection				
Branch to Clade 1	<0.001***	0.207	<0.001***	
*A. japonicum*	0.005**	**0.049**	0.268	
Branch to *C. am* and *T. per*	<0.001***	NA	0.196	
*T. perfoliata*	<0.001***	<0.001***	1.000	
Branch to *C. am*	0.001**	NA	1.000	
*C. americana App*	0.160	1.000	1.000	
*C. americana West*	0.023*	1.000	0.859	

Only genes/gene families with evidence for episodic diversifying selection across the full tree, excluding outgroups, were included. aBSREL was only run on Clade 1 when BUSTED found a signature of episodic diversifying selection for this clade. Values in bold were significant at p < 0.05.

**Figure 5:**
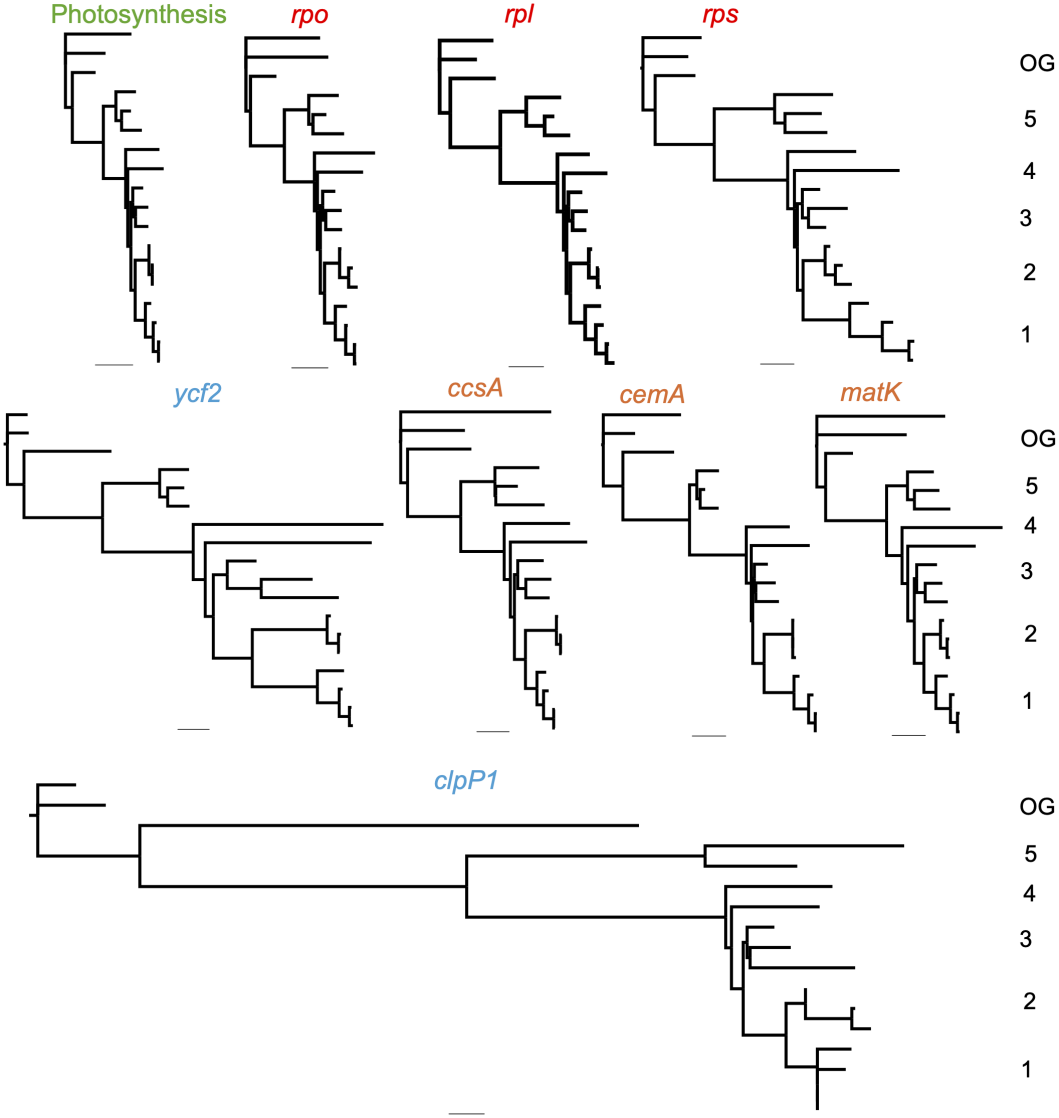
Rates of evolution of plastid genes across the Campanulaceae from a phylogenetic perspective. Branch lengths for each gene/gene concatenation were estimated on a constrained tree topology, the same topology used for the dN/dS analyses. All photosynthetic genes were combined into a single concatenation due to high similarities. Green = photosynthetic; Red = gene regulation; Blue = proteostasis, Orange = other. Clades are labelled as in **Figure 1**, with *C. americana* in clade 1. Trees were visualized and standardized by scale using FigTree (v1.4.4) such that the scale bar is equivalent across trees, and branch lengths are directly comparable.

Examining plastid gene evolution using phylogenetic trees yielded similar patterns, with the *rps* and *ycf2* trees having longer branch lengths than most other plastid genes (**Figure 5**). In particular, the greatest rates of evolution for the focal clade (1), were found in the *rps* gene tree. However, the longest branch lengths were found in the *clpP1* tree, which also exhibited the highest variability, though the *clpP1* sequence was identical across the *C. americana* lineages.

## Discussion

Rates of evolution and signatures of selection varied widely among plastid genes for *C. americana* and across the Campanulaceae. Photosynthetic genes exhibited lower rates of nucleotide substitution and low dN/dS ratios indicative of purifying selection, while elevated rates and dN/dS ratios were primarily found in genes involved with plastid gene regulation and proteostasis, in particular *clpP*, *rps*, and *ycf2*. However, distinct patterns of evolution were observed for each of these genes.

Within *C. americana*, dN/dS ratios well above one were observed for only one gene family, the small ribosomal proteins (*rps*), with values of 2.16 and 3.33 for Appalachian and Western lineages respectively. These high dN/dS ratios strongly suggest these genes are under positive selection. BUSTED and aBSREL results further supported positive selection acting on *rps* genes within *C. americana*, with significant results found for *C. americana* as a whole and on the branch leading to the Western, though not Appalachian lineage. Interestingly, these dN/dS ratios are much higher than was found in a previous study that used only the Appalachian lineage (dN/dS = 0.92; Barnard-Kubow et al., 2014), indicating the importance of including both lineages (Appalachian and Western) and their sister species (*Triodanis perfoliata*) to obtain insight into the recent evolutionary dynamics of these plastid genes. A dN/dS ratio above one was also found for *ycf2* in the Western lineage (dN/dS = 1.18), similar to that observed previously (dN/dS = 1.09). However, it was uncalculatable in the Appalachian lineage due to a dS of zero. In contrast to *rps*, no support was found for positive selection acting on *ycf2* within *C. americana*. Two additional genes, *ycf1* and *clpP1*, exhibited dN/dS ratios above one in the previous study but were uncalculatable in the current study for different reasons. The *ycf1* gene was so highly divergent across all 18 species that a reasonable alignment was unattainable, while the *C. americana clpP1* sequences were identical.

When expanding the focus to across the Campanulaceae, a similar set of genes exhibited patterns indicative of positive selection, including dN/dS ratios above one for genes related to plastid gene regulation and proteostasis. However, distinct patterns of evolution were observed within these two gene categories. The *rps* genes only exhibited dN/dS ratios close to or above one in the two *C. americana* lineages and closely related species in clades one and two, suggesting a recent acceleration in these clades. Recent acceleration is further supported by the fact that clade 1 exhibits longer branches in the *rps* gene tree than any other tree and the fact that the best two rate branch model in PAML supported an elevated dN/dS in clades 1 and 2 relative to the remaining taxa. In contrast, genes involved with proteostasis, both *clpP1* and *ycf2*, exhibited dN/dS ratios above one in species spread across the Campanulaceae including the outgroup in the family sister to the Campanulaceae (*Carpodetus serratus*). These two genes also showed the greatest rates of evolution when examining branch lengths on a constrained tree topology, with multiple long internal branches, further indicating both historic and ongoing accelerated evolution.

Taking into account patterns of plastid gene evolution within *C. americana* and across the Campanulaceae, the *rps* genes are the strongest candidate plastid genes for PNI in *C. americana*. These genes exhibited a dN/dS ratio well above one in both *C. americana* lineages, evidence of positive selection in the focal clade and within *C. americana*, and increased rates of sequence evolution in the focal clade relative to the rest of the Campanulaceae. The *rps* genes also showed elevated levels of sequence divergence when looking across multiple *C. americana* populations (López-Caamal et al., 2025). Elevated rates of nucleotide substitution and dN/dS ratios have been found for plastid ribosomal genes in multiple taxa with rapidly evolving plastid genomes (Guisinger et al., 2008; Postel et al., 2022; Sloan et al., 2014b), and coevolution of nuclear-encoded subunits has also been documented (Postel et al., 2022; Sloan et al., 2014b), leading to the potential for mismatch and PNI when crossing between genetic lineages. One of these taxa, *Silene nutans*, exhibits a PNI phenotype similar to *C. americana*, and a disruption of the plastid ribosome has been suggested as a contributor (Postel et al., 2023; Postel et al., 2022).

The 12 plastid-encoded *rps* genes make up the small subunit of the plastid ribosome, along with 12 nuclear-encoded subunits and the plastid-encoded 16S rRNA (Tiller and Bock, 2014; Yamaguchi et al., 2000). The plastid ribosome translates all plastid-encoded gene transcripts and thus is essential for plastid function and photosynthesis. Mutations eliminating function of plastid ribosomal genes in rice lead to an albino phenotype similar to the PNI phenotype in *C. americana* (Gong et al., 2013; Lin et al., 2015; Zhou et al., 2021). However, mutation of ribosomal plastid genes in *Arabidopsis* and *Nicotiana* often results in embryo lethality (Meinke, 2020; Tiller and Bock, 2014). This difference may be due to whether the plastid genome contains (or lacks) the *accD* gene which encodes a subunit of the heteromeric ACCase required for fatty acid biosynthesis (Parker et al., 2014). In rice and other Poaceae, plastids lack *accD* and instead use a nuclear-encoded, plastid targeted homomeric ACCase. Interestingly, in *C. americana*, and the Campanulaceae as a whole, *accD* has been moved to the nuclear genome (Rousseau-Gueutin et al., 2013), which could potentially lead *C. americana* to behave similarly to rice.

Two other genes exhibited striking patterns of evolution across the Campanulaceae but are less promising candidates for the PNI in *C. americana*. One of these, *clpP1*, had dN/dS ratios above one for most species and exhibited the greatest rates of evolution across the Campanulaceae. This finding fits with the highly variable and at times rapid rates of evolution of *clpP1* across angiosperms (Erixon and Oxelman, 2008; Rockenbach et al., 2016; Williams et al., 2022; Williams et al., 2019). Compensatory evolution of the nuclear-encoded *clpP* subunits in response to plastid substitutions has also been documented (Forsythe et al., 2021; Rockenbach et al., 2016), indicating that the *clpP* complex could be prone to disruption of cytonuclear coevolution when crossing between divergent lineages, making this a potentially good candidate for PNI. However, within the Campanulaceae, the longest branches on the *clpP1* gene tree are internal branches leading to the Campanulaceae, specifically to clade 5 and to the ancestor of clades 1-4. Thus, much of the accelerated evolution in this gene is likely historical in the family. This fits with the pattern of accelerated rates of evolution in *clpP1* prior to the split of *C. americana* and *Trachelium caeruleum* (clade 4) but subsequent reduced rates found in the earlier study (Barnard-Kubow et al., 2014). Finally, the *clpP1* sequence was identical between the Western and Appalachian *C. americana* lineages, consistent with low sequence divergence for this gene found in previous work in *C. americana* (López-Caamal et al., 2025). Taken together, these results do not support a role for the *clpP* complex in PNI in *C. americana*, although it has the potential to play a role in other taxa.

As with *clpP1*, *ycf2* exhibited elevated rates of evolution and dN/dS ratios above one in multiple species across the Campanulaceae, including the Western lineage of *C. americana*. The *ycf2* gene encodes a subunit of the Ycf2-FtsHi complex, which acts as the motor of the TOC-TIC complex responsible for translocating nuclear-encoded preproteins into the chloroplast (Kikuchi et al., 2018; Liang et al., 2024). *Ycf2* acts a scaffold, facilitating assembly of the Ycf2-FtsHi complex by stabilizing the other components, all of which are nuclear encoded, and acting as a pivotal interaction hub (Liang et al., 2024; Xing et al., 2025). Interestingly, *ycf1* (Tic214), which was dropped from analysis in this study due to high levels of sequence divergence, is thought to play a similar scaffold role in the TIC complex (translocon of the inner envelope of the chloroplast). *Ycf1* and *ycf2* are the largest genes in the plastid genome, are essential for cell survival (Drescher et al., 2000) and in the case of *ycf1* can vary widely in sequence length among species (Xing et al., 2025). These two genes also frequently exhibit increased rates of diversity and nucleotide substitution, and in some cases elevated dN/dS ratios, indicating they are dynamically evolving (Dong et al., 2015; Greiner et al., 2008; Lin et al., 2025; Moghaddam et al., 2022; Neubig et al., 2009; Sloan et al., 2012).

Protein import is an essential process for plastids, and mutants knocking out the function of proteins involved in these complexes frequently are embryo lethal or exhibit albinism (Drescher et al., 2000; Kikuchi et al., 2018; Kikuchi et al., 2013). Thus, *ycf1* and *ycf2* could be good candidate genes for the plastid component of PNI in *C. americana*, as mismatches between these plastid-encoded genes and the nuclear-encoded subunits could lead to the phenotypes observed. Further support for a role of *ycf1* and/or *ycf2* comes from previous work identifying elevated levels of sequence divergence for these genes in *C. americana* (López-Caamal et al., 2025). However, the dN/dS ratio is only moderately above one in just one *C. americana* lineage and there was no evidence for positive selection acting on *ycf2* in most of the focal clade or within *C. americana*. Thus, the elevated dN/dS observed in the western *C. americana* lineage is more likely due to relaxed selection than positive selection, Therefore, while involvement of *ycf2* and *ycf1* in the PNI in *C. americana* cannot be excluded, the evidence is overall weaker than for the *rps* genes.

In contrast to the elevated dN/dS ratios observed for multiple genes/gene families involved with gene regulation and proteostasis, plastid genes related to photosynthesis consistently showed low dN/dS values both within *C. americana* and across the Campanulaceae. These low values indicate consistent purifying selection acting on photosynthetic genes. Similar results have been observed in other studies of species with rapid plastid evolution, where elevated dN/dS ratios are typically found in non-photosynthetic genes and photosynthetic genes exhibit high sequence conservation and low dN/dS (Greiner et al., 2008; Lin et al., 2025; Moghaddam et al., 2022; Postel et al., 2022; Sloan et al., 2012). These results indicate that photosynthetic genes are likely already highly optimized and sequence level changes are not favored, suggesting that cytonuclear coevolution of photosynthetic protein complexes is unlikely to be a common mechanism underlying PNI. Regulation of plastid photosynthetic genes, however, is a different story, and has been shown to contribute to PNI in *Oenothera* (Zupok et al., 2021).

Overall, the small ribosomal proteins (*rps*) appear to be the most promising candidate plastid genes for the PNI in *C. americana*, potentially leading to a loss or reduction of plastid translation. However, a contribution of *ycf1* and *ycf2* and a corresponding impact on plastid protein import cannot be ruled out. In addition, analyses are based only on plastid gene sequences. Future work on analysis of dN/dS in nuclear-encoded, plastid-targeted genes, in particular the components of the small ribosomal, TIC and Ycf2-FtsHi complexes will enable direct testing for cytonuclear coevolution in these protein complexes. If found, this would confirm results presented here and provide definitive evidence for the genes and mechanisms underlying PNI in *C. americana*.

Beyond *C. americana*, future work should investigate possible PNI in Campanulaceae taxa for which currently there is no information. In particular, results suggest evaluating possible PNI in other clade 1 and clade 2 species that exhibit elevated sequence evolution and dN/dS for the *rps* genes. It would be particularly interesting to test for PNI in *Adenophora divaricata* that has elevated rates of evolution and dN/dS across multiple plastid gene families. Finally, taxa from other Campanulaceae clades might show alternative patterns of PNI related to *ycf2* or *clpP*, or perhaps no PNI at all. An assessment of PNI across the Campanulaceae would help determine to what extent accelerated plastid evolution and subsequent plastid-nuclear coevolution could act as a driver of speciation and diversification in this family.

## Data Availability

The datasets presented in this study can be found in online repositories. The names of the repository/repositories and accession number(s) can be found below: https://github.com/kbkubow/CampanulaceaePlastidGeneEvolution.
